# Why Women Get so Short of Breath

**Published:** 1889-08

**Authors:** D. A. Sargent


### WHY WOMEN GET SO SHORT OF BREATH.

In order to ascertain the influence of tight clothing upon the action of the heart during exercise, a dozen yonng women consented, this summer, to run 540 yards in their loose gymnasium garments, and then run the same distance with corsets on. The running time was two minutes and thirty seconds for each person at each trial ; and, in order that there should be no cardiac excitement or depression following the first test, the second trial was made the following day. Before beginning the running, the average heart impulse was 84 beats to the minute. After running the above named distance, the heart impulse was 152 beats to the minute, the average natural waist girth being 25 inches. The next day, corsets were worn during the exercise, and the average girth of waist was reduced to 24 inches. The same distance was run in the same time by all ; and, immediately afterward, the average heart impulse was found to be 168 beats per minute. When I state that I should feel myself justified in advising an athlete not to enter a running or rowing race whose heart impulse was 160 beats per minute after a little exercise, even though there were not the slightest evidence of disease, one can form some idea of the wear and tear on this important organ, and the physiological loss entailed upon the system in women who force it to labor for over half their lives under such disadvantage as the tight corset imposes.—DR. D. A. SARGENT, in *Scribner's.*

